# Cricothyrotomy - In Unanticipated Difficult Intubation Cases with Respiratory Compromise

**DOI:** 10.1055/s-0043-1776726

**Published:** 2024-01-24

**Authors:** S Sathiyabama

**Affiliations:** 1 Department of ENT, PSG Institute of Medical Sciences and Research, Coimbatore, Tamil Nadu, India

**Keywords:** intubation, tracheostomy, airway management, respiration, oxygen saturation

## Abstract

**Introduction** Cricothyrotomy, percutaneous dilation tracheostomy, and tracheostomy are all cost-effective and safe techniques used in the management of critically ill patients who need an artificial airway other than endotracheal tube ventilation. The present study focused on enlightening on elective and emergency procedures performed on conditions present with difficult airways and also attempts to shed light on the aspects of securing an airway in anticipated and unanticipated difficult intubation.

**Objective** The objective of the study was to compare the three procedures conducted during difficult airway/failed intubation situations.

**Methods** The present retrospective observational study was conducted collecting data from patient files obtained at a tertiary healthcare center from 2013 to 2018. The difficult intubation cases were managed by ear, nose, and throat (ENT) surgeons. The study compared three methods: Cricothyrotomy, percutaneous dilation tracheostomy, and tracheostomy based on factors such as procedure duration, complications, and the instruments required for each procedure.

**Results** The study enrolled 85 patients, 61 males and 24 females, aged between 30 and 70 years old. To perform cricothyrotomy, only a simple blade was required. Cricothyrotomy had the shortest operating time (4.1±3.1 minutes) and the shortest time of full oxygen saturation (3 min). Percutaneous tracheostomy had the least amount of bleeding (1%). Cricothyrotomy significantly showed the least intraoperative bleeding than percutaneous dilation, tracheostomy, and tracheostomy ( *p* = 0.001).

**Conclusion** Cricothyrotomy is preferable as it takes less time to perform, causes less bleeding, and takes the least time for full oxygen saturation than tracheostomy and percutaneous dilatational tracheostomy in “can't intubate, can't oxygenate” patients.

## Introduction

Intubation difficulties are a common issue that anesthesiologists face daily in the operating room. 1 Difficult airways are more common in patients undergoing ear, nose, and throat (ENT) surgery. Intensive care unit (ICU)-related tracheal intubations are potentially hazardous primarily due to failing oxygenation and unstable hemodynamics while performing emergency intubations. 2 In both urgent and emergency cases, ENT surgeons and anesthesiologists frequently collaborate to perform surgeries. 3 When there is any respiratory difficulty for an unconscious patient, mechanical ventilation is to be established by the intensive care doctors. 4

If the intubation fails, it can result in catastrophic damage to patients, including brain damage or death. Hence, difficult airway management in hospital settings are tended to by a team of anesthesiologists and otolaryngologists. In nondifficult airway management, anesthesiologists alone can manage with endotracheal tube for ventilation. However, the role of ENT surgeons is significant to establish airway in both cases of anticipated or unanticipated difficult airway.

Otolaryngologists usually attend the call from the emergency department to perform elective tracheostomy on intubated patients to reduce ventilator associated pneumonia (VAP) score. 5 However, unanticipated difficult intubation failure can result in rapid sequence induction and respiratory compromise for which emergency surgical intervention by an Otolaryngologist will be needed. 6 Tracheostomy ventilation is a standard procedure in cases of upper airway obstruction and other methods like percutaneous tracheostomy and cricothyrotomy can also be used with limited indications.

Depending on the definition used, the prevalence of difficult intubation ranges from 0.1 to 10.1%. 1 A previously conducted study reported that the incidence of patients who suffered from difficult tracheal intubation events between 2000 to 2012 was 23% in nonperioperative locations. 7 Another study that assessed 4,000 reports to the webAIRS anesthesia incident reporting database reported 170 incidents of difficult or failed intubations. 8

Tracheostomy is one of the ancient and widely used procedures in critically ill patients. Percutaneous dilatational tracheostomy is renowned as the standard of care in the ICU and has largely replaced surgical tracheostomy in this subset of patients. 9 High frequency jet ventilation, surgical or needle cricothyrotomy, supraglottic airways, and cardiopulmonary bypass are the initial rescue measures in difficult airway conditions. 10 Emergency cricothyrotomy is the final step in the “Can't intubate, can't oxygenate” (CICO) algorithm and is performed to prevent the patient's death. Complication rates vary by study, depending on the level of training, clinical scenario, or location of the procedure, and can range from 0 to 54%. 11 Preintubation ultrasound has been considered for increasing the rate of successful cricothyrotomies. 12 However, there is no gold standard for a difficult airway algorithm. 3 The primary objective of the present study is to compare and evaluate all three methods in terms of indications, duration of operating time, intraoperative and postoperative complications, and the instruments needed for each procedure.

## Methodology

The present retrospective study was carried out at a tertiary healthcare center located in Coimbatore, Tamil Nadu, India, between January 2013 and January 2018. Data was collected from case sheets obtained from the medical record section of the emergency department and ICU. Prior to initiation of the study, ethical clearance was obtained from the institutional ethics committee (PSG/IHEC/2020/App/Exp/234). All study procedures were conducted by trained otolaryngologists employed at the hospital.

**Study population:** The total number of cases examined was 582 and, from these, 85 patients suffered from difficult intubations or failed intubations and needed an airway for further management and these patients were included in the present study. Patients with easy ventilation with endotracheal tubes without tracheostomy were excluded from the study.

### Interventions

The present study compared three methods, tracheostomy, 13 percutaneous dilatational tracheostomy, 14 and cricothyrotomy 15 for factors such as duration of the procedure, complications (earlier and late), and instruments needed for each procedure. All three procedures were performed by an otolaryngologist during the emergency calls. All calls were attended by different ENT surgeons.

All suspected cases of difficult airway underwent fiberoptic laryngoscopic examination. First, nonsurgical techniques like intubation were performed. If two consecutive attempts failed, oxygen saturation was maintained by means of face mask ventilation until an airway was established through surgical technique/intervention. The instruments used and the time taken for each procedure were described in Table 1 .

**Table 1:** Comparison between different procedures

Method	Instruments	Operating time
Tracheostomy	Special set of instruments includes knife, monopolar cautery, langenberg retractor, tracheal dilator, alice forceps	10-15 minutes
Percutaneous tracheostomy	Separate kit which includes a 22-gauge needle and syringe; 11-F short punch dilator; 1.32-mm 28-F, 32-F, 36-F, and 38-F dilators; Shiley no.6 tracheostomy tube guidewire; 8-F guiding catheter; 18-F, 21-F, 24-F,	10-15 minutes
Cricothyrotomy	Scalpel or 15 blade. Tracheostomy tube 6 size	1-3 minutes

According to the 2016 American Society of Anesthesiologists (ASA) physical status classification system practice guidelines, the anesthesiologist should have a preplanned strategy for intubation of the difficult airway. 16 Surgical technique can be the best option to be considered for difficult intubation cases as it is a lifesaving procedure and ENT surgeons should have expertise in proper selection of the technique to be employed and resultant success at establishing airway in failed intubation cases.

The two procedures that were conducted as part of the study are:

**Tracheostomy** : 13 The tracheostomy procedure was first documented in history around 1546 B.C. for peritonsillar abscess cases to relieve resultant airway obstruction. Except for cases of impending airway obstruction, tracheostomy is performed on an elective basis for patients who require long-time ventilation. It is an opening in the anterior wall of the trachea holding the tracheostomy tube for an alternate pathway of breathing in case of individuals with upper airway obstructions. Usually in ICUs, early transition to tracheostomy (ETT) is a procedure recommended by anesthesiologists in order to reduce the incidence of VAP. This emergency procedure is performed under local anesthesia on patients exhibiting supraglottic obstruction with stridor. Ideally, the tracheostoma should lie over the 2 ^nd^ to the 3 ^rd^ tracheal ring cartilages. High tracheostomy can lead to subglottic stenosis and low tracheostomy can result in possible injury to the pleura. Since this is an open procedure, it is essential to avoid complications, and this is easily manageable. The vertical incision is made 2 to 3 cm inferior to the cricoid, which makes it unlikely that we encounter any vascular structures. Skin and strap muscles are retracted, the isthmus of the thyroid is lifted by a hook, and the anterior tracheal wall is incised after which the tracheostomy tube is inserted through the opening. Cuffed tubes are ideal for mechanical ventilation to facilitate positive pressure ventilation.

**Percutaneous tracheostomy** : 14 The challenges of airway establishment also depend upon the operating room time and on the burden of transporting critically ill patients, which was the impetus behind developing a quick, safe, and reliable alternative to open tracheostomy. Toye et al. 17 first described percutaneous tracheostomy using the Seldinger technique in 1969, and remained in practice until Ciaglia 18 introduced the dilatational percutaneous technique in 1985. The surgeon should recognize that all patients are good candidates for PDT but it is contraindicated in children because the trachea is collapsible, mobile, and difficult to localize for safe performance. 19 It has also been reported as a contraindication in children according to a study by Kost as well as to a review conducted by Cho. 20 21 Obese individuals should also be managed with special care as thyroid swelling is also a contraindication for this procedure. The most commonly used technique for PDT was first described by Ciaglia et al., which was a bedside technique in which the guide wire was passed between the 2 ^nd^ and 3 ^rd^ tracheal rings. Sequential dilatation using graduated dilators over the guidewire created a passage through which the tracheostomy tube is placed. 22 Since it is a form of blind procedure, the chances of posterior tracheal wall injury are present but can be prevented by adopting videobronchoscopic guidance as needed. 23

**Cricothyrotomy** : 15 Difficult airway sentinel events occur in critical care departments due to lack of coordination between specialty providers, difficulty in access to equipment to address the airway management at bedside and unavailability of training/experience on the respective procedure. Only ENT surgeons can perform this procedure with minimal requirement of instrumentation.

Despite the introduction of numerous rescue devices for failed airway, the most common errors in the management of difficult airway are the result of repeated and consecutive unsuccessful attempts at intubation. Once identified as a CICO situation, immediate cricothyrotomy is the ideal surgical intervention to be performed by an otolaryngologist and should be practiced accordingly. In order to prevent hypoxic brain injury, early airway establishment by this technique is to be considered as the best option, hence, it is a highly recommend practice. However, the main obstacle is certainly lack of experience or failure in recognizing the timely need to perform this procedure.

The following are the important rapid five steps of the technique: 15

1. Extend the neck and identify the thyroid angle and fix the cartilage.
2. Make a vertical skin incision with a 15G blade after palpating the lower border of the thyroid cartilage and extend 2 mm below it.
3. The blade should be inserted perpendicularly through the cricothyroid membrane (CTM).
4. Insert a clamp to spread and elevate the airway.
5. Insert a smaller sized tracheostomy tube or a smaller sized ETT and immediately inflate the cuff

The anatomical structures to be considered during the procedure include:

**Surgical anatomy of the cricothyroid membrane:**^24^ The CT) is bordered superiorly by the thyroid cartilage, inferiorly by the cricoid cartilage, and laterally by the bilateral cricothyroideus muscles. It is a fibroelastic triangular membrane. Its upper border is free and stretches between the thyroid angle to the vocal process of the arytenoids and forms the vocal ligament. Its lower border attaches to the arch of the cricoid cartilage.

**Dimensions of the cricothyroid membrane** : 25 In an average adult male, it is 1 cm in length and 2 to 3 cm wide and is in the midline about a fingerbreadth below the laryngeal prominence. The vocal folds are ∼ 1 cm above the CTM and therefore prone to injury during this procedure. The ligament does not calcify. The anterior part of the conus elasticus is the middle cricothyroid ligament (ligamentum cricothyreoideum medium; central part of the cricothyroid membrane). It is thick and strong, narrow above and broad below. It connects the front parts of the contiguous margins of the thyroid and cricoid cartilages. It is overlapped on either side by the cricothyreoideus, but subcutaneous in between; it is crossed horizontally by a small anastomotic arterial arch, formed by the junction of the two cricothyroid arteries, whose branches pierce it.

**Vascular anatomy of the cricothyroid membrane** : 26 The cricothyroid artery crosses the upper one-half of the cricothyroid membrane. Branches of the cricothyroid artery penetrated the membrane and ascended along the under surface of the thyroid cartilage. The superior thyroid artery coursed anterior to the sternothyroid muscle and then the lateral edge of the cricothyroid membrane. The membrane was also crossed by venous tributaries to the superior and inferior thyroid veins.

### Instruments for Various Procedures:

- Conventional tracheostomy: Tracheostomy instrument set including tracheal dilator, artery forceps, and retractors.
- Percutaneous tracheostomy: Separate special kit, video bronchoscope
- Cricothyrotomy: Scalpel, artery forceps, cuffed ETT, or tracheostomy Tube

## Result

**Fig. 1:**
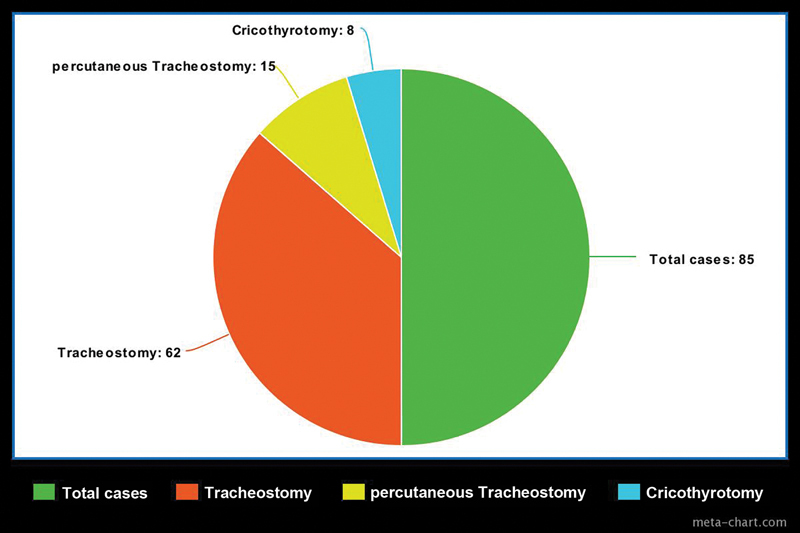
Distribution of patients for three different procedures.

The study recruited 85 patients, consisting of 61 males and 24 females, with an age ranging between 30 and 70 years old. Fig. 1 illustrates the distribution of patients for three different procedures. Most participants consisted of unconscious ICU patients. Table 1 elaborates the comparison of instruments needed for each procedure and the time required. To perform cricothyrotomy, only a simple blade was required. The procedure was completed within 1 to 3 minutes, which was the least compared to tracheostomy and percutaneous tracheostomy. However, to perform tracheostomy and percutaneous tracheostomy, special kits were used and the time taken to complete these procedures was from 10 to 15 minutes.

Table 2 illustrates the comparison of different procedures based on operating time, intraoperative complications, and time of full oxygen saturation. The operating time (4.1 ± 3.1minutes) and time of full oxygen saturation (3 minutes) were observed in cricothyrotomy. The least bleeding was observed in percutaneous tracheostomy (1%).

**Table 2:** Comparison of different procedures (based on operating time, intraoperative complication, and time of full oxygen saturation)

	Operating time	Intra-operative complication	Time of full oxygen saturation
**Conventional tracheostomy**	12.78 ± 3.18 minutes	• Bleeding 3% • Subcutaneous emphysema 2%	10-15 minutes
**Percutaneous tracheostomy**	18.38 ± 3.3 minutes	• Bleeding 1% • False track 2%	10-15 minutes
**Cricothyrotomy**	4.1 ± 3.1 minutes	• Bleeding 3%	3 minutes

Table 3 depicts a comparison of different procedures based on intraoperative bleeding. The intraoperative bleeding was graded from 0 to 5. It was noted that cricothyrotomy significantly showed the least intraoperative bleeding than percutaneous dilation tracheostomy and tracheostomy (t = ,12.588664; *p* < 0.001).

**Table 3:** Comparison of different procedures (based on intraoperative bleeding)

	Intraoperative bleeding (Grade: 0–5)	t-value	*p* - *value*
**Conventional tracheostomy**	1.4 ± 1.04	12.588664	< 0.001
**Percutaneous tracheostomy**	3.5 ± 0.9		
**Cricothyrotomy**	3.0 ± 0.5		

Table 4 shows that 10 patients who underwent tracheostomy suffered from subcutaneous emphysema, 4 patients who underwent percutaneous tracheostomy underwent severe bleeding, and 1 patient who underwent cricothyrotomy suffered from moderate bleeding. Tracheostomy and percutaneous tracheostomy took 10 to 15 minutes to provide access to airway while cricothyrotomy took 3 minutes.

**Table 4:** Analysis of complications

Activity	Complication	Time to access airway
Tracheostomy	Subcutaneous emphysema – 10 patients	10-15 minutes
Percutaneous tracheostomy	Severe bleeding – 4 patients	10-15 minutes
Cricothyrotomy	Moderate bleeding – 1 patient	3 minutes

## Discussion

Cricothyrotomy and percutaneous dilatational tracheostomy placement are important means of securing an artificial airway in patients with acute or chronic respiratory failure. 27 Despite advances in airway management techniques and refinement of difficulty predictors, the cited 1 to 3% unanticipated difficulty has not been changed, making the role of the otolaryngologist essential. In a quest to determine which procedure can be used for a difficult airway, the present study aims to focus on enlightening the elective and emergency procedures performed for difficult airway conditions. To the best of our knowledge, this is the only study that compared all three procedures and evaluated all three methods in terms of indications, duration of operating time, intraoperative and postoperative complications, and the instruments needed for each procedure.

Data from 85 patients were evaluated, out of which the majority of the patients were males (71.1%). The age of the patients ranged between 30 and 70 years old. This was aligned with Wnent et al., who also reported that the predictive factors for impossible intubation were male gender and younger age. 28 Endlich et al *.* reported that the prevalence of difficult and failed airway intubation incidents mostly occurred in patients aged between 40 and 59 years old. 8

Out of 85 patients, the maximum number of patients underwent conventional tracheostomy. This may be because cricothyrotomy and percutaneous dilatational tracheostomy procedures are expensive compared to conventional tracheostomy. Moreover, these procedures require a lot of skill development. 29

Percutaneous tracheostomy and cricothyrotomy cases were low in number to assess its significance. This was due to patients suffering from cervical spine injuries being difficult to shift to the operation theatre; patients who underwent percutaneous tracheostomy were under high level of respiratory support. Cricothyrotomy was done in situations where intubation and oxygenation were not possible in order to improve saturation with quick access. The knowledge of cricothyrotomy and adequate training are key factors for the success of this procedure.

Four of the patients who underwent percutaneous tracheostomy died due to comorbidities, with the remaining 11 patients undergoing successful decannulation. All cricothyrotomy patients were changed to conventional tracheostomy after successful airway access on the same day and the cricothyrotomy wound was closed. Postoperative follow-up was done for 1 year, no subglottic stenosis was noticed, and successful decannulation was done for 7 patients. One patient went against medical advice.

The present study also compared the instrument and the time taken to complete the procedure by three methods: cricothyrotomy, percutaneous dilation, and tracheostomy. The simplest and easily available instruments that were used among the three procedures were scalpel or 15 blades for cricothyrotomy, and it can take only 1 to 3 minutes to complete the procedure. Technically, an emergent cricothyroidotomy is not a difficult procedure. However, it may be prohibitive to the unprepared team from a cognitive standpoint. 11 On the other hand, percutaneous tracheostomy requires a separate kit requiring a gauge needle and syringe, short punch dilator, Shiley no.6 tracheostomy tube, guide wire, and guiding catheter. It takes almost 10 to 15 min to complete the procedure. Percutaneous tracheostomy is a safe and cost-effective procedure used in patients who require prolonged mechanical ventilation, most commonly in the ICU. 27

In the present study, cricothyrotomy took the least operating time (4.1 ± 3.1minutes). In the previous study conducted by Anasuya et al *.* , to perform a cricothyroidotomy procedure, it took 75 to 180 seconds *.* A cricothyrotomy procedure would need a simple blade; as a result, it can be completed without wasting time. 30 The time of full oxygen saturation reported for cricothyrotomy was 3 minutes. A study by Chen et al *.* showed that SpO2 was increased postoperatively, reaching 95% at 12 seconds, 131 seconds, and 144 seconds in the cricothyroid membrane puncture guided tracheostomy (CMPGT), surgical cricothyroidotomy (SC) and Griggs guidewire dilating forceps (GWDF) groups, respectively. 31

Among all aforementioned procedures, the least amount of intraoperative bleeding was noted in cricothyrotomy, and this was assessed by calculating the number of soaked gauzes. For most patients who require an emergency surgical airway, cricothyrotomy is preferable to tracheostomy because it is easier to perform, causes less bleeding, and takes less time to perform than an emergency tracheostomy. 11 The only obstacle is the lack of experience or timely recognition of the need to perform this procedure. 29

The advantages and features of cricothyrotomy over other procedures are that no major vessels intervene in the cricothyroid membrane area, the localization is easy in patients with thin necks, it can be performed in obese patients by sliding the finger from the thyroid angle and feeling the lower border of the cartilage in the midline, rapid entry, and placing the tracheostomy tube and inflating the cuff immediately will prevent both bleeding and subcutaneous emphysema.

The limitation of the present study was the retrospective nature of the study which can result in information bias. Another limitation was that cerebral hypoxia improvement was not assessed or compared. The operating surgeon was a confounding factor, although it was reduced by including a surgeon with the same level of straining and experience in all procedures. Males outnumbered females in all groups. Although it was difficult to exactly estimate the amount of intraoperative blood loss, we adopted the surgical field method as well as graded the intraoperative bleeding both reported by a surgeon for assessment of imperative bleeding. Future studies should aim to include a large number of patients and should include a detailed postoperative review.

## Conclusion

All three procedures *,* tracheostomy, percutaneous dilatational tracheostomy, and cricothyrotomy, can be used for maintaining airway procedure in various conditions. However, our study recommended tracheostomy and percutaneous tracheostomy as suitable techniques in difficult intubations but with oxygen saturation maintained with an oxygen mask. In desaturating and difficult cases that represent the CICO scenario, cricothyrotomy is the gold standard procedure, as it takes less time to perform, causes less bleeding, and takes the least time for full oxygen saturation than tracheostomy and percutaneous dilatational tracheostomy.
